# Thanatophoric Skeletal Dysplasia: A Case Report

**DOI:** 10.31729/jnma.4488

**Published:** 2020-03-31

**Authors:** Firoz Anjum, Sunil Kumar Daha, Ganesh Shah

**Affiliations:** 1 Department of Pediatrics, Patan Academy of Health Sciences, School of Medicine Patan Hospital, Lalitpur, Nepal

**Keywords:** *birth defects*, *micromelia*, *thanatophoric dysplasia*

## Abstract

Thanatophoric skeletal dysplasiais the most lethal, rare, sporadic birth defect due to de novo mutation in the fibroblast growth factor receptor-3. Clinically this is characterized by shortening of the limbs (micromelia), small conical thorax, flat vertebral bodies and macrocephaly at birth. We encountered a similar case with ultrasonographic findings suggestive of Thanatophoric Skeletal Dysplasia which resulted in the death of the baby within an hour of birth. Almost all cases of this condition have been reported to have died interuterinally or a few days after birth.

## INTRODUCTION

Skeletal dysplasia refers to a heterogeneous group of heritable disorders characterized by abnormalities of cartilage and bone growth, resulting in abnormal shape and size of skeleton and disproportion of long bones, spine and head.^1^ Thanatophoric Skeletal Dysplasia is the most lethal, rare, sporadic birth defect due to de novo mutation in the fibroblast growth factor receptor-3 (FGFR3).^2,3,4,5,6,7^ We encountered a newborn baby with shortening of the limbs (micromelia), small conical thorax, flat vertebral bodies and macrocephaly and ultrasonographic findings suggestive of TSD.Because of very high mortality and no case report from Nepal, we aimed to report this case to make health professionals more aware of this rare condition.

## CASE REPORT

**Figure 1:**
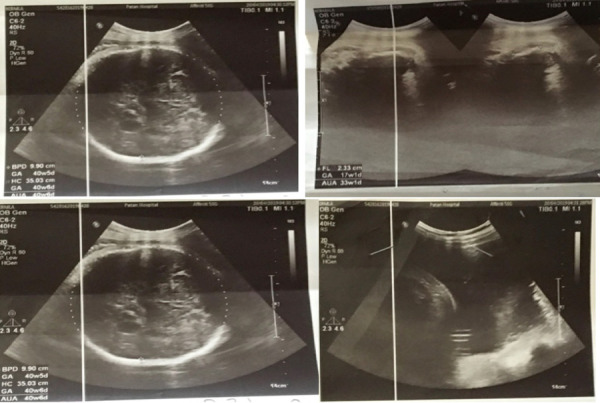
USG showing features suggestive of TSD.

A 22 years old non-smoker, non-alcoholic primigravida who had a normal regular antenatal check-up and no chronic illnesses or family history of congenital abnormality came to Patan Hospital for her routine checkup at 39 weeks of gestation (WOG).USG was done revealing average age of fetusas 39 plus weeks and femoral length of 17 weeks of gestation (2.3 cm) only, biparietal diameter 9.9cm (corresponding to 40 WOG), head circumference was 35.2cm (corresponding to 41 WOG), Arm circumference 34.1cm (corresponding to 38 WOG). All four limbs were small to corresponding gestation (micromelia) and Amniotic Fluid Index (AFI) 30 cm which is suggestive of Thanatophoric Skeletal Dysplasia (Figure 1).

The emergency lower segment cesarean section (LSCS) was done at 39+6 WOG for fetal bradycardia and macrocephaly. A single, live, male with a birth weight of 3270 grams was born. The Apgar scorewas 4/10 in the first minute and 3/10 in the fifth minute. The baby did not cry immediately after birth despite rigorousstimulation and suctioning. We started bag and mask ventilation and still, there was no cry and heart rate was less than 60 beats per minute which were gradually decreasing. Meanwhile, the need for ventilator support and Neonatal Intensive Care Unit (NICU) care was counseled to the patient's family but they denied doing any lifesaving interventions and the baby died after an hour of birth.

**Figure 2:**
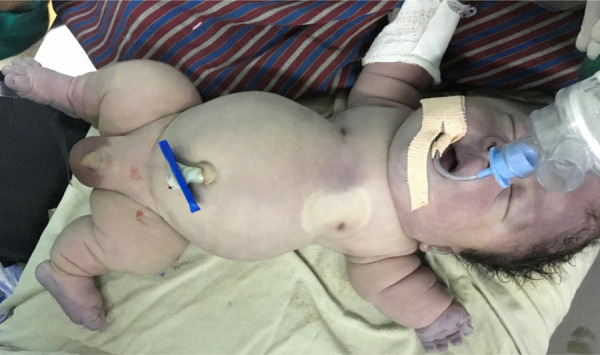
Showing micromelia, macrocephaly, narrow thorax, and protruding abdomen.

**Figure 3:**
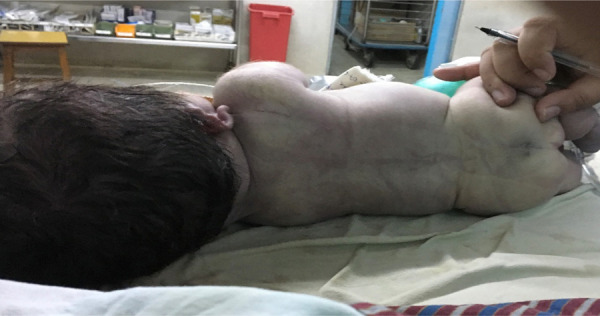
Showing flat back and spine with pit/ dimpling over the sacral region.

Examination revealedthe length of 36 cm (<5th centile for newborn boy),macrocephaly (OFC: 38 cm,inbetween 90th and 95th percentile), frontal bossing, short neck, central and peripheral cyanosis, saddle nose, low set ears. The sutures were not separated. The thorax was narrow, cone-shaped with small rib cage and abdomen was protuberant with the abdominal girth of 33 cm. All the limbs were small with a deformed attitude (Lower segment: 14 cm, Upper segment 22 cm and the ratio was 1.57). Thighs and legs were bowed. Deep creases were present in all limbs (Figure 2 and Figure 3).

There was small pit/dimpling over the sacral region, cryptorchidism and flat back and spine (Figure 3). However, there were no murmurs, passed meconium immediately after birth and the umbilical cord has 2 arteries and one vein.

## DISCUSSION

TSD is the most lethal, rare, sporadic birth defect due to de novo mutation in the FGFR3 gene.^3,4^ FGFR3 gene is located in chromosome 4p16.3, responsiblefor givinginstructions for making a protein that is involved in the development and maintenance of bone and brain tissue.^4^ The malformations due to bone growth seen in TSD are due to overactivation of FGFR3 gene.^5^ The examination findings, in this case, revealsmacrocephaly, narrow thorax associated with polyhydramnios, micromelia, bowed thigh, frontal bossing, saddle nose, low set ears, protruding abdomen and flattening of the spine. We found similar findings in the different reported case of type I TSD.2-7 The radiological and morphological features revealed in our report (Figure 1) confirms the diagnosis of Type I TSD.2-7 Phenotypically TSD is of two clinically defined subtypes: Type I and Type II, the former being the more frequent (80%).^4^

The inheritance pattern of TSD is autosomal dominant but virtually all cases of TSD occur in people with no family history of TSD.^6^ The reason being, no affected individuals are known to have had children.Therefore, the disorder has not been passed to the next generation. As presented in our case, TSD usually leads to death inutero or shortly after birth.^5,6,7,8,9,10^ The reason for mortality in TSD is either due to reduced thoracic capacity, hypoplastic lung or brainstem compression.^6,7,8^

Usually, the diagnosis of TSD is made by USG duringthe second trimester and further specific type is distinguished on later scans during the third trimester with the help of fetal skeletal morphology.10 Further diagnosis can be confirmed with autopsy and histopathology but unfortunately could not be done in the present case as consent was not given by the parents.^2,3,3,5,6,7,8,9,10^

The radiologic and morphologic features described in this report were compatible with TSD Type I. These findings helped us to correlate them with their pathogenesis and realize the reason why this disease usually has a poor prognosis. Proper counseling to the patient's family is crucial for management and should be advised to undergo anomalies screening in subsequent pregnancies.

## Consent:

**JNMA Case Report Consent Form** was signed by the patient and the original article is attached with the patient's chart.

## Conflict of Interest

**None.**
